# Do Microplastics in Soil Influence the Bioavailability of Sulfamethoxazole to Plants?

**DOI:** 10.3390/plants14111639

**Published:** 2025-05-27

**Authors:** Anna Parus, Natalia Lisiecka, Arkadiusz Kloziński, Joanna Zembrzuska

**Affiliations:** 1 Institute of Chemical Technology and Engineering, Poznan University of Technology, Berdychowo 4, 60-965 Poznan, Poland; natalia.lisiecka@doctorate.put.poznan.pl (N.L.); arkadiusz.klozinski@put.poznan.pl (A.K.); 2 Institute of Chemistry and Technical Electrochemistry, Poznan University of Technology, Berdychowo 4, 60-965 Poznan, Poland; joanna.zembrzuska@put.poznan.pl

**Keywords:** accumulation, antibiotic, migration, phytotoxicity, soil pollution, xenobiotics

## Abstract

The presence of pharmaceuticals and MPs in soil raises concern due to their potential impact on plant health and ecosystem stability. This study investigates the impact of MPs on the bioavailability and phytotoxicity of sulfamethoxazole (SMX) using sorghum as a model plant. Three types of MPs—polyethylene (PE), polystyrene (PS), and acrylonitrile–butadiene–styrene copolymer (ABS)—were analyzed in primary and aged forms. The results indicate that MPs influence SMX sorption and desorption, affecting its bioavailability in soil. Low SMX concentrations (≤5 mg/kg) stimulated sorghum growth, while higher concentrations (≥25 mg/kg) significantly inhibited germination and biomass production. The presence of 1% MPs in soil generally reduced SMX toxicity, suggesting a role for MPs in modifying antibiotic availability in the soil matrix. Bioavailability analyses confirmed interactions between MPs and SMX or MPS and soil components, with variability depending on polymer type and ageing process. Among the tested MPs, polystyrene showed the strongest effect on increasing SMX bioavailability in both primary and aged forms. These findings highlight the environmental implications of MPs in agricultural soils, particularly concerning contamination, crop quality, and antibiotic resistance. A deeper understanding of MP–pharmaceutical interactions is crucial for evaluating long-term ecological risks and formulating effective mitigation strategies.

## 1. Introduction

In recent decades, increasing attention has been paid to the widespread accumulation of pharmaceuticals in the environment. The widespread production and use of pharmaceuticals result in their continuous release into both aquatic and terrestrial environments [1,2]. Several pharmaceuticals, such as antibiotics, antidepressants and beta-blockers, have been detected in environmental samples, both in soil and surface water [3].

Sulfamethoxazole (SMX) is a widely used sulfonamide antibiotic applied in both human and veterinary medicine, most often in combination with trimethoprim. Due to its extensive use, SMX is frequently detected in rivers, lakes, groundwater, and soil. Its primary entry routes into the environment include municipal and hospital wastewater, industrial discharges, and agricultural activities—particularly through manure application and the use of contaminated irrigation water [4,5,6,7]. Standard wastewater treatment plants often fail to completely remove SMX, resulting in its persistence in aquatic and terrestrial systems. Although some studies report susceptibility of SMX to photodegradation and biodegradation, it remains relatively resistant in deeper soil and water layers, leading to long-term accumulation [8]. Environmental concentrations of SMX vary depending on population density, industrial activity, and land use, ranging from a few to several hundred ng/L in water and up to 100 ng/g in fertilized topsoil [1,3,9,10,11]. SMX is considered highly mobile in soil due to its relatively low sorption affinity, especially in mineral soils with low organic content [12,13]. Its sorption behaviour is strongly influenced by soil pH, with the molecule existing in cationic form under acidic conditions and in neutral or anionic forms at higher pH values. These forms differ in their interaction with soil particles and influence the degree of SMX mobility and bioavailability. Numerous studies have demonstrated that SMX can exert phytotoxic effects, including reduced germination, inhibited root elongation, impaired photosynthesis, and oxidative stress responses in various plant species [14,15,16]. These findings highlight the environmental relevance of SMX contamination and the importance of assessing its fate and effects in soil–plant systems.

Microplastics (MPs) are another important environmental factor under extensive investigation. Polystyrene (PS), acrylonitrile butadiene styrene (ABS), polyethylene (PE), and other polymers can enter soil from various sources. Their emission into the environment is mainly related to human activity, waste degradation, and industrial processes. Among other things, PE-based agricultural films are widely used in crop production to protect soil and retain moisture; their fragmentation contributes to MPs introduction into the soil. Certain fertilizers and plant protection products may contain microplastics as carriers for active ingredients. Additionally, treated sewage sludge used as fertilizer may contain PS, ABS, and PE particles originating from industrial and domestic sources. Inadequately secured landfill sites can also cause microplastics to enter the soil [17,18,19].

Previous research has shown that microplastics can alter the environmental fate of antibiotics such as sulfamethoxazole (SMX). For example, Guo et al. [20] and Yu et al. [21] reported that various microplastic types—particularly PE and polyamide (PA)—are capable of adsorbing SMX under environmentally relevant conditions. Additional studies by Zhang et al. [22], Wang et al. [23], and Ren et al. [24] confirm that MPs can reduce SMX bioavailability by modifying its sorption–desorption dynamics in soil. These findings support the hypothesis that microplastics may reduce the mobility and toxicity of SMX by functioning as passive sorbents or carriers.

Studies suggest that MPs can alter soil structure by creating additional spaces that influence water retention or drainage. Additionally, MPs can interact with other contaminants, promoting their retention, accumulation, and decreased bioavailability, thereby prolonging their environmental persistence. MPs also undergo weathering processes, causing surface defects, fragmentation, and sometimes even chemical structural changes. This can result in the formation of additional sorption sites, which further enhance interactions with other pollutants [17,18,19].

Due to these factors, both SMX and MPs pose significant environmental challenges, particularly regarding their long-term presence in soil and their potential impact on organisms and the development of antibiotic resistance. Therefore, this study investigates how the presence of MPs (PE, PS, and ABS) influences SMX bioaccumulation and toxicity in model plants, with particular emphasis on the mechanisms of sorption and desorption. The novelty of this study lies in identifying how the type and ageing of MPs affect SMX bioavailability and linking these changes to plant-level phytotoxic responses, thereby providing an integrated view of contaminant mobility and biological impact in soil systems.

## 2. Results

### 2.1. Determination of the Effects of MPs and SMX Interactions on Germination and Early Plant Development

**Figure 1:**
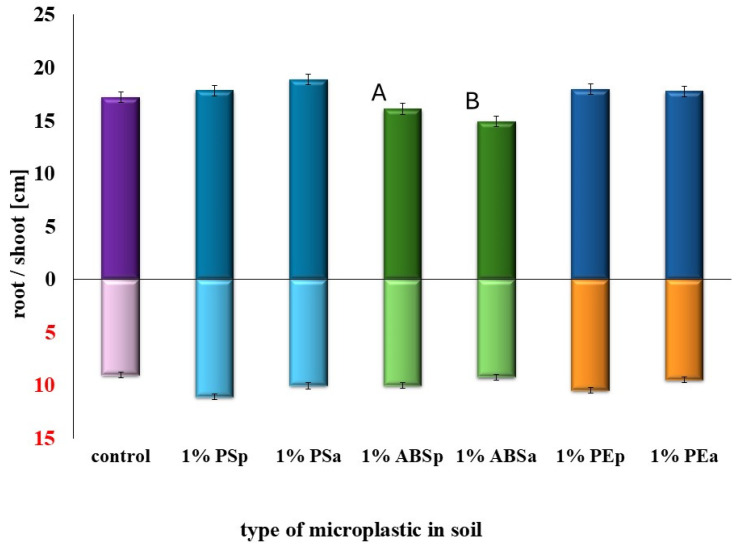
MPs influence on germination and growth of sorghum (p—primary, a—aged). Different letters indicate statistically significant differences between groups (*p* < 0.05).

The results indicated that the analyzed microplastics (MPs), regardless of their type (PE, PS, ABS) or ageing state (primary or aged), did not negatively affect the germination percentage or early growth parameters of sorghum (Figure 1). In all MP treatment variants, germination rates remained comparable to the control. The shoot and root lengths also showed no significant reduction, except for the highest concentration of ABS, where a slight but measurable decrease in shoot length was observed—approximately 10% compared to the control (*p* < 0.05).

**Figure 2:**
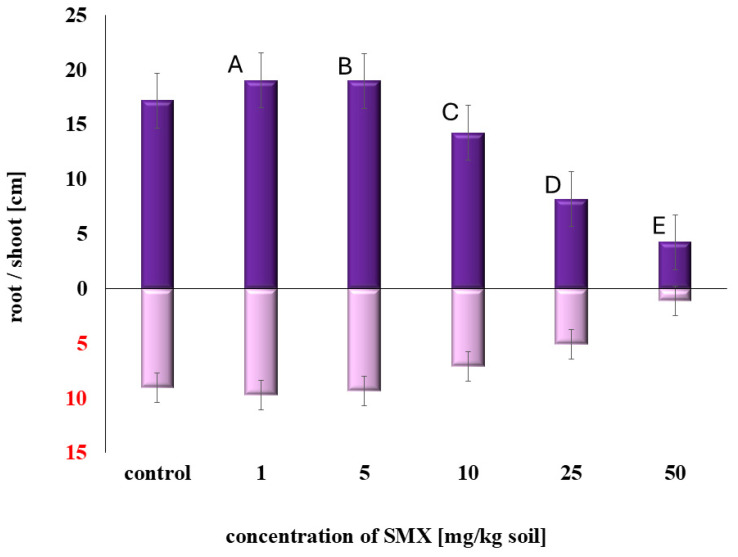
The impact of SMX on sorghum growth. Different letters indicate statistically significant differences between groups (*p* < 0.05).

In contrast, increasing concentrations of SMX had a clear inhibitory effect on plant development (Figure 2). At lower doses (1–5 mg/kg), SMX slightly stimulated root and shoot elongation, though the differences were not statistically significant. However, at 10 mg/kg, sorghum growth was visibly inhibited, with shoot length reduced by approximately 25% relative to the control. At 25 mg/kg, both germination and growth were reduced by around 50%. At the highest tested concentration (50 mg/kg), root elongation was almost entirely suppressed, and seedling emergence was significantly delayed or absent in most replicates.

### 2.2. Analysis of the Impact of Microplastics on Changes in SMX Toxicity

**Figure 3:**
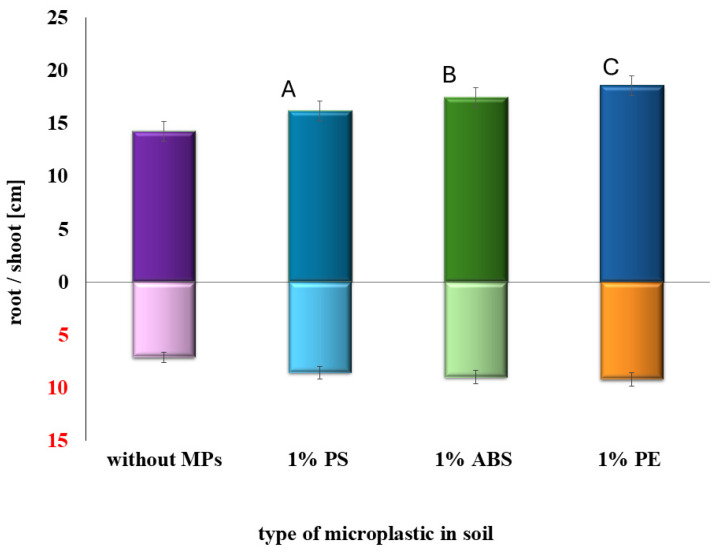
Impact of MPs on SMX toxicity change (tests performed at EC25). Different letters indicate statistically significant differences between groups (*p* < 0.05).

**Figure 4:**
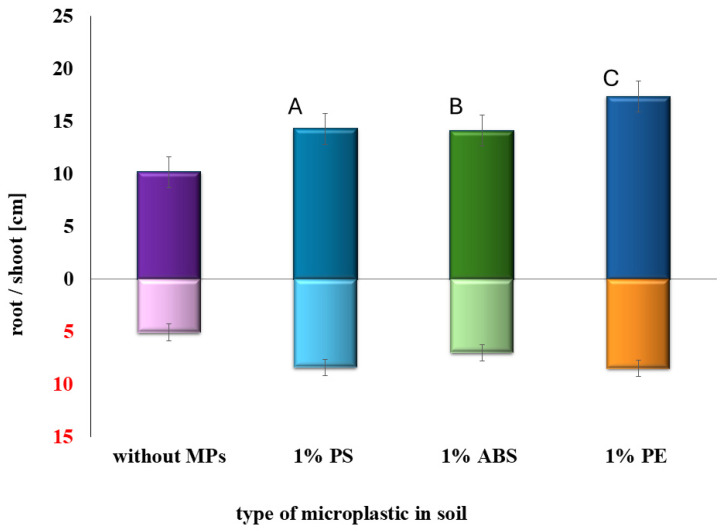
Impact of MPs on SMX toxicity change (tests performed at EC50). Different letters indicate statistically significant differences between groups (*p* < 0.05).

In the main stage of the study, aged MPs were used to evaluate their influence on SMX toxicity. Figure 3 and Figure 4 show that the addition of 1% MP to SMX-contaminated soil improved sorghum growth compared to soil treated with SMX alone. This trend was observed for all tested MP types. The presence of MPs resulted in longer shoots and roots, and increased germination percentage in both EC25 and EC50 systems. Among the tested polymers, polystyrene appeared to produce the most pronounced increase in shoot and root length. In all MP-amended systems, plants exhibited significantly better development compared to the corresponding controls without MPs (*p* < 0.05).

### 2.3. Change in Accumulation of SMX Due to Presence of MPs in Soil

The sorption and desorption of SMX in soil were analyzed based on the concentrations of SMX in eluates obtained after the two-step BCR extraction (Table 1). The results showed that the addition of microplastics to the soil did not significantly influence the total amount of SMX retained in the solid phase. Similar sorption values were recorded for soil with and without MPs. However, differences were observed in SMX concentrations measured in the eluates, depending on the polymer type and whether the MPs were primary or aged. The highest SMX concentrations in the eluates were found in soil systems containing primary PE and ABS microplastics. In contrast, eluate concentrations in soil with aged MPs were similar to or slightly lower than those in the control without MPs.

## 3. Discussion

Understanding the interactions between MPs and pharmaceuticals in soil is critical for assessing their combined effects on plant development, contaminant mobility, and ecological risk.

The phytotoxicity tests confirmed that SMX exhibits dose-dependent effects on sorghum. At lower concentrations (1–5 mg/kg), SMX slightly stimulated shoot and root growth, while higher doses (≥10 mg/kg) significantly inhibited development, with nearly complete suppression of root elongation at 50 mg/kg. These results are consistent with previous studies documenting SMX phytotoxicity in species such as *Lemna gibba*, *Brassica juncea*, and *Allium cepa* [14,15,16].

MPs can alter the environmental behaviour of antibiotics. Previous studies showed that PE and PA particles adsorb SMX under environmental conditions [20,21] and may reduce its bioavailability in soil [22,23,24]. This can impact the concentration of freely available antibiotics in the soil solution and, consequently, plant exposure levels.

In our study, the addition of MPs to SMX-contaminated soil resulted in improved plant development compared to SMX alone. This observation is consistent with reports showing that even ionic liquid-based herbicides caused minimal morphological changes in plants under certain conditions [25]. Zhou et al. [26] demonstrated that MPs may increase cadmium bioavailability in soil, while similar desorption-enhancing effects have been observed in biosurfactant-amended petroleum-contaminated soils [27]. Liu et al. [28] also confirmed that MPs can alter contaminant desorption dynamics in complex matrices.

The lack of phytotoxicity from MPs alone aligns with previous studies. Liwarska-Bizukojc [29] reported no inhibitory effects of PLA on sorghum, while Zhang et al. [30] and Roy and Gerson [31] showed no significant root or shoot effects in plants grown with PS or PE. Comparable biphasic responses, including low-dose stimulation and high-dose inhibition, have been observed with rhamnolipids and ionic liquids in *Lemna minor* and mustard plants [32,33].

High concentrations of SMX suppressed plant growth in agreement with previous findings. Our earlier study showed that SMX increases metal bioavailability in soil [34], and similar results were observed in rice, rapeseed, and maize exposed to antibiotics [35,36,37,38]. This inhibitory effect may involve oxidative stress, as SMX exposure has been shown to disrupt antioxidant activity and increase ROS production in rapeseed [39,40].

MP-induced changes in root morphology, SMX uptake, and transpiration were also reported in lettuce grown in PP-contaminated soil [41,42,43]. MPs may influence soil porosity and moisture retention, thereby affecting root access to water and contaminants [44,45].

Moreover, MPs affect the structure and function of microbial communities. They can promote microbial colonization and biofilm formation, which in turn influences SMX degradation rates [46,47,48,49]. These microbial interactions may contribute to the decreased SMX toxicity observed in MPs-amended soils.

Our findings also support the role of MP ageing in determining their environmental function. Aged MPs, with higher surface roughness and oxygenated functional groups, showed greater retention of SMX than primary MPs, which have smoother, less polar surfaces [50,51,52,53,54].

The observed behaviour of SMX in soil–MPs systems may also be linked to its chemical speciation and charge state. SMX exists in cationic, neutral, or anionic forms depending on soil pH, which influences its interaction with both MPs and mineral particles [12,55,56]. These interactions are fundamental to understanding its sorption, mobility, and plant availability.

Furthermore, the combined presence of MPs and SMX may pose complex risks to terrestrial organisms beyond plants. Studies have shown that such mixtures can affect soil enzyme activity, nematode populations, and the behaviour of invertebrates [57,58,59,60]. These findings highlight the importance of considering MP–xenobiotic interactions in ecological risk assessments.

## 4. Materials and Methods

### 4.1. Microplastics

The materials used in this study included acrylonitrile–butadiene–styrene copolymer (ABS), polystyrene (PS), and polyethylene (PE). Both ABS and PS were obtained under the trade name Terluran GP-35 (INEOS Styrolution Group GmbH, Frankfurt, Germany) and were cryogenically ground using a ZM 200 mill equipped with a DR 100 vibratory feeder (Retsch, Katowice, Poland). Grinding was carried out in the presence of dry ice to prevent polymer plasticization and improve fragmentation efficiency, following the protocol reported by Lisiecka et al. [61], which also includes the physicochemical characteristics of PS.

In contrast, micro-PE was purchased as a ready-made material from Sigma Aldrich (St. Louis, MO, USA), and its properties have been detailed in Lisiecka et al. [62].

In addition to the primary materials, aged microplastics were also employed in the study. Ageing was conducted using a modified Fenton protocol, simulating environmental oxidative conditions under laboratory settings, as described by Lisiecka et al. [63], which also provides a full physicochemical characterization of ABS microplastics.

FT-IR analyses confirmed that no new functional groups were introduced during ageing. Key parameters such as surface area (BET), average pore diameter, total pore volume, and mean particle size are summarized in Table 2.

### 4.2. Soil Characteristics

The OECD soil used in this study consisted of 70% air-dried quartz sand, 20% kaolin clay, and 10% peat. Calcium carbonate was used to obtain an initial pH of 6 ± 0.5 (in KCl) and 6.5 (in water). The water holding capacity was 40%, the total carbon was 5%, and the cation exchange capacity was 8.76 cmol/kg. Data represent the mean of triplicate soil analysis (standard error of mean < ± 5%, *n* = 3).

### 4.3. Assessing the Effects of MPs and SMX Interactions on Germination and Early Plant Development

The effect of MPs and SMX on the initial growth of the model plant and the seed germination process was evaluated in accordance with OECD Guideline No. 206, as described in detail in study [64]. Sorghum (Sorghum) seeds were used as the model plant.

The study was divided into two stages. The first stage focused on the individual effects of MPs and SMX on sorghum germination. Soil was amended with 0.1%, 0.5%, and 1% of individual MPs (both primary and aged), with concentrations selected based on average levels commonly used in plant growth studies involving microplastics [65,66]. SMX was introduced into the soil at concentrations of 1, 5, 10, 20, and 50 mg/kg dry soil weight (d.s.w.). Water-irrigated soil without SMX or MPs served as the control. After one week, the lengths of shoots and roots were measured, and the number of germinated seeds was recorded. Germination indexes were calculated in accordance with the methodology described in the study [67].

Based on the results of the first stage, EC25 and EC50 values for SMX were determined (according to the methodology described in [50]) and subsequently applied in the second stage of the study. This stage focused on evaluating interactions between SMX and MPs, and their impact on SMX bioavailability, germination, and sorghum development. For this purpose, 1% of individual aged MPs were added to the soil alongside SMX at EC25 or EC50 concentrations. Soil containing SMX at these concentrations but without MPs was used as a reference. After sowing the seeds, cultivation was carried out in the same manner as in the first stage, with measurements taken after seven days. Phytotoxicity tests were performed three times for all analyzed systems in both stages of the study to ensure the repeatability of the results.

### 4.4. Analysis of the Effect of MPs on Changes in SMX Accumulation

The change in SMX accumulation in the MP-contaminated soil was analyzed by assessing sorption and potential bioavailability.

The effect of MPs on SMX sorption in soil was analyzed in a manner analogous to that described in the study [32] and in accordance with OECD guidelines [68]. The experiments were conducted using soil with a 1% addition of MPs (both primary and aged) and a control sample—soil without MPs. Since the aim of the study was to analyze changes in the antibiotic’s bioavailability under the influence of MPs, the experiment was conducted for two SMX concentrations corresponding to EC25 and EC50. The process was carried out in glass flasks for 24 h. After this period, the samples were centrifuged, filtered through a PTFE syringe filter, and analyzed using HPLC-MS to determine the amount of SMX in the supernatant.

Changes in SMX bioavailability in soil contaminated with MPs were analyzed using the first two sequential extraction steps (BCR). This procedure was based on the methodology described in the study [50], which provided important information on the effect of MPs on the amount of SMX available in the soil.

For the experiment, SMX was added to a 10 g soil sample at concentrations of 10 and 25 mg/kg soil dry weight, i.e., corresponding to EC25 and EC50. The samples were mixed thoroughly and dried at 60 °C until a constant mass was obtained.

In a first step, the amount of water-soluble SMX was determined. For this purpose, 1 g of soil sample was placed in 50 mL glass centrifuge tubes, 40 mL of double-distilled water was added, and the samples were shaken (280 rpm, 16 h, 20 °C). After this process, the samples were centrifuged (10,000 rpm, 10 min), the resulting supernatants were filtered through a PTFE syringe filter (0.22 µm) and transferred to new containers.

In the second extraction step, 40 mL of acetic acid solution (0.11 mol/L) was added to the residue from the first step, and an identical procedure was performed. The collected supernatants from both steps were filtered through a PTFE syringe filter and analyzed for SMX content by HPLC-MS/MS.

### 4.5. HPLC-MS Analysis

SMX was determined based on ultrahigh-performance liquid chromatography coupled with tandem mass spectrometry (LC-MS/MS) using an UltiMate 3000 RSLC liquid chromatograph (Dionex, Sunnyvale, CA, USA) connected with an API 4000 QTRAP mass spectrometer (Biosystem, MDS Sciex, Framingham, MA, USA) as described by Parus et al. [32]. Five µL samples were injected into a 100 mm × 2.1 mm I.D. analytical column packed with 1.9 µm Hypersil Gold C18 RP from Thermo Scientific (Waltham, MA, USA). In the experiments, the column was kept at 35 °C, and the mobile phase consisted of 0.1% formic acid (A) and acetonitrile (B) at a flow rate of 0.2 mL/min. Isocratic elution was used (A:B ratio, 4:6).

The API 4000 QTRAP triple quadrupole mass spectrometer from AB Sciex (Toronto, ON, Canada) performed the analyte’s detection. The Turbo Ion Spray source operated in positive ion mode. The dwell time for mass transition detected in the MS/MS multiple reaction monitoring mode (MRM) was set at 70 ms. All the studies were detected using the following settings for the ion source and mass spectrometer: curtain gas 20 psi, nebulizer gas 40 psi, auxiliary gas 45 psi, temperature 600 °C, ion spray voltage 5500 V, and collision gas set to medium. The MS/MS parameters used for the quantitative determination of SMX are presented in Table 3. [M+H]^+^ complexes of tested SMX were used as precursor ions.

The concentrations of all analytes were determined using the standard curve technique.

### 4.6. Statistical Analysis

Statistical analysis of the data was performed using one-way analysis of variance (ANOVA) (with *p* < 0.05), which is appropriate for comparing means across multiple independent treatment groups. Normality and variance homogeneity were verified; Tukey’s HSD test was applied post hoc.

## 5. Conclusions

This study demonstrates that microplastics can influence the phytotoxicity and environmental behaviour of sulfamethoxazole (SMX) in soil. While SMX alone exhibited a dose-dependent inhibition of sorghum growth, the addition of microplastics—particularly aged ones—mitigated these effects. MPs did not impair plant development on their own and, in combination with SMX, improved germination and growth parameters compared to SMX-only treatments.

Sorption analysis revealed that the presence of microplastics in soil modified SMX phytotoxicity, with co-application generally resulting in improved plant performance compared to SMX alone. This suggests that MPs influence the mobility and plant-accessible fraction of SMX in the soil matrix.

These findings underscore the complex role of microplastics in regulating antibiotic mobility and toxicity in soil–plant systems. They also highlight the importance of considering MP–contaminant interactions in environmental risk assessment frameworks. Future studies should address long-term effects under field conditions and evaluate the combined impact on soil microbiota, plant productivity, and food safety.

## Figures and Tables

**Table 1:** Influence of MPS on sorption and bioavailability of SMX in soil (p—primary, a—aged).

Type of Soil	Adsorption [%]	Bioavailability [%]	Adsorption [%]	Bioavailability [%]
	10 mg/kg Soil (EC25)		25 mg/kg Soil (EC50)	
OECD	26.9 ± 0.2	88.9 ± 0.5	25.1 ± 0.2	85.1± 0.6
OECD + ABSp	23.2 ± 0.4	86.3 ± 0.6	23.2 ± 0.3	94.1 ± 0.9
OECD + ABSa	28.0 ± 0.9	84.4 ± 0.4	27.6 ± 0.1	90.4 ± 0.5
OECD + PSp	22.5 ± 0.1	99.5 ± 0.3	23.4 ± 0.2	104.1 ± 0.8
OECD + PSa	27.3 ± 0.3	89.2 ± 0.4	29.7 ± 0.3	96.8 ± 0.7
OECD + PEp	21.0 ± 0.5	99.4 ± 0.8	19.4 ± 0.4	101.1 ± 0.3
OECD + PEa	21.6 ± 0.2	88.1 ± 0.9	21.1 ± 0.3	86.4 ± 0.2

**Table 2:** Physicochemical characteristics of primary (p) and aged (a) microplastics used in the study.

Type of MPs	BET Surface Area [m^2^/g]	Average Pore Diameter [nm]	Total Pore Volume [cm^3^/g]	Mean Particle Size [μm]
ABSp	0.2	15.3	0.001	242
ABSa	0.3	14.4	0.001	242
PSp	0.2	23.1	0.001	350
PSa	0.1	23.9	0.001	350
PEp	0.2	18.6	0.001	500
PEa	0.2	21.0	0.001	500

**Table 3:** MS/MS parameters for the acquisition of SMX.

Compound	Precursor Ion [M-H]–m/z	Declustering Potential (V)	MRM1 */MRM2 ** Transitions Ion (Precursor Ion m/z → Product Ion m/z)	Collision Energy (V)
SMX	291	11	291 → 230 291 → 123	33 35

* for quantitation, ** for confirmation.

## Data Availability

The metadata have been deposited in the RepOD repository and can be accessed via the link https://doi.org/10.18150/LYETW1.

## Abbreviations

The following abbreviations are used in this manuscript:

MPs	Microplastics
SMX	Sulfamethoxazole
PE	Polyethylene
PS	Polystyrene
ABS	Acrylonitrile–Butadiene–Styrene Copolymer
PLA	Polylactic Acid
ROS	Reactive Oxygen Species
CAT	Catalase
APX	Ascorbate Peroxidase
EC25	Effective Concentration Causing 25% Inhibition
EC50	Effective Concentration Causing 50% Inhibition
d.s.w.	Dry Soil Weight
